# Histomorphology, Sperm Quality and Hormonal Profile in Adult Male Sprague-Dawley Rats following administration of aqueous crude Extract of *Solanum nigrum* by gastric gavage

**DOI:** 10.5935/1518-0557.20180051

**Published:** 2018

**Authors:** Sunday Aderemi Adelakun, Babatunde Ogunlade, Toluwase Solomon Olawuyi, Julius Akomaye Aniah, Olusegun Dare Omotoso

**Affiliations:** 1 Department of Human Anatomy, School of Health and Health Technology, Federal University of Technology, Akure, Ondo State, Nigeria; 2 Department of Anatomy, College of Medicine, University of Abuja, Federal Capital Territory (FCT), Nigeria; 3 Department of Anatomy, Faculty of Basic Medical Sciences, Kogi State University Anyigba, Kogi State, Nigeria

**Keywords:** Solanum nigrum, fertility, luteinizing hormone, testosterone, sperm count, sperm motility

## Abstract

**Objective:**

The present study focused on histomorphology, sperm quality, hormonal profile and hematological parameters in adult male Sprague-Dawley rats following the administration of aqueous crude extract of *Solanum nigrum* by gastric gavage.

**Methods:**

Fourty healthy male adult (12-14 weeks old) Sprague-Dawley rats weighing 200-220g were randomly divided into four groups (A,B,C and D) of ten (n=10) rats each. Group A which served as control were given distilled water 2ml/kg b.wt each, daily for 28 days. Group B, C and D rats were administered 100, 300 and 500mg/kg b.wt each daily respectively for 28 days. The extract was saved with LD_50_ >5000mg/Kg. Sperm counts, percentage motility, morphology and percentage live sperm, hormonal profile and hematological parameter were quantified; testis, epididymal and general body weights were measured using a weighing scale. The extract was administered once daily for six days within a week via oral gavage. After the last administration, all rats were sacrificed by cervical dislocation, the testis were harvested and fixed in Bouin‘s fluid for histology processing.

**Results:**

Our results revealed an increase in sperm counts, percentage of motility, morphology and percentage of live sperm, blood level of follicle stimulating hormone, Luteinizing hormone and testosterone, hematological parameters, testis, epididymal and general body weights across the groups in a dose-depentant manner. The testis histoarchtecture showed normal cellular composition in their germinal epithelium, with sperm cells in the lumen and a normal interstitium.

**Conclusion:**

This experiment revealed that aqueous extract of *Solanum nigrum* bears profertility properties which may be beneficial to those who consume it.

## INTRODUCTION

*Solanum nigrum* is a vegetable, as well as a herbal plant found in southwest Nigeria and most parts of the world. Nightshade also known as "odu" (*Solanum nigrum*) is an annual plant. It is commonly consumed as cooked complement to some major staple food like cocoyam, cassava, yam etc. (Ajala, 2009). It is also a common plant found in most parts of Europe and the African continent (Atanu *et al*., 2011). Various epidemiological studies reported that *Solanum nigrum* protects against various ailments (Jain *et al.,* 2011). It has been used traditionally for the treatment of bacterial infections, cough, indigestion, hepatitis, pain, inflammation and fever (Zakaria *et al*., 2006; Lee & Lim, 2003). The plant is also used in the Oriental systems of medicine for various purposes, such as antiproliferative (Li *et al.,* 2009; Nawab *et al.,* 2012), antiseizure (Wannang *et al.,* 2008), antioxidant (Lee & Lim, 2003), antiviral (Javed *et al.,* 2011), anti-inflammatory (Kang *et al.,* 2011) and hepatoprotective effects (Lin *et al.,* 2008; Hsieh *et al*., 2008). It has been reported that the *Solanum nigrum* extract’s biological activity may vary based on its extraction method (Das *et al.,* 2010). Water extracts of *Solanum nigrum* have been shown to contain active compounds such as tannins, alkaloids, phytosterols, flavonoids and coumarins (Ravi *et al.,* 2009). Different phytochemicals in *Solanum nigrum* have different effects. It has been shown to be protective in carbon-tetrachloride-induced liver damage in rats (Lin *et al.,* 2008); inhibit thioacetamide-induced liver fibrosis in mice (Hsieh *et al.,* 2008); cytoprotective in gentamicin-induced kidney cell damage *in vitro* (Kumar *et al.,* 2001) and also in preventing trypanosome-induced liver damage thus increasing the survival time of mice infected with *T. b. rhodesiense* (Serem *et al.,* 2013).

In many countries of the world, green leafy vegetables are used for food, being a rich source of ß-carotene, ascorbic acid, minerals and dietary fiber (Sun *et al.,* 2002; Oboh, 2005; Oboh & Akindahunsi, 2004; Oboh & Rocha, 2007). The potential of the Nigerian flora as a veritable source for pharmaceuticals and other therapeutic materials has been emphasized (Gbile & Adesina, 1986). Apart from healing, these leafy vegetables provide the necessary nutrients for a healthy development of the human body. In the past, the average African rural dweller depended on subsistence farming in which he cultivated vegetable crops for his immediate family consumption (Ayodele, 2005). Vegetables are also known to be an important source of protective foods and occupy a major place among food crops. They provide adequate amounts of many vitamins and minerals for humans. Studies have shown that apart from lower methionine content, the amino acid profile of leaf species compare favorably with those of soya bean, fish and egg (Agbaire & Emoyan, 2012).

The present study focused on evaluating histomorphology, sperm quality, hormone profile and hematological parameters in adult male Sprague-Dawley rats following administration of aqueous crude extract of *Solanum nigrum* by gastric gavage.

## MATERIALS AND METHODS

### Plant Material

The plant materials were collected from the Research Farm, School of Agricultural Sciences, Ladoke Akintola University of Technology (LAUTECH) Ogbomoso, Oyo State, Nigeria in April, 2016. The *Solanum nigrum* samples were identified and authenticated by Prof. A.J. Ogunkunle of the Department of Pure and Applied Biology, and a sample of the plant voucher was deposited for reference purposes.

### Extraction of plant material

The leaves were thoroughly washed in sterile water and air-dried to a constant weight in the laboratory. The air-dried leaves were weighed using a CAMRY (EK5055, Indian) - electronic weighing balance and were milled in an automatic electrical blender (model FS-323, China) to powdered form. Five hundred grams of the milled plant sample was later soaked in 1000 ml of Phosphate buffered saline (PBS) for 48 hours (Iweala & Okeke, 2005) at room temperature, and was later filtered through cheese cloth and then through Whatman #1 filter paper (Khan *et al*., 2010). The filtrate was concentrated using a rotary evaporator (Rotavapor^®^ R-210) at 42-47ºC.

### Phytochemical screening

Phytochemical analysis of the aqueous leaf extract of *Solanum nigrum* was done qualitatively and quantitatively, in accordance with Soni & Sosa (2013). High performance liquid chromatography was adopted to quantify the vitamins by modifications of the report by Grindberg & Williams (2010). Minerals content such as sodium, calcium, Potassium, iron, zinc and phosphorus were determined using modification of the method described by Akubugwo *et al*. (2008).

### Acute Toxicity Studies

The acute toxicity studies (LD_50_) of Solanum *nigrum* was determined using the Lorke method (1983). The study was carried out in two phases. In the first phase, 9 rats were used. The rats were randomly divided into three groups, with 3 rats in each group. Group 1 received 10mg/Kg, group 2 received 100mg/Kg and group 3 received 1000mg/Kg via oral route respectively, and observed for signs of toxicity and death for 24 hours. In the second phase, 4 rats were used, consisting of 4 groups with a rat in each group. Group 1 received 1000mg/Kg, group 2 received 1600mg/Kg, group 3 received 2900mg/Kg and group 4 received 5000mg/Kg. The median lethal dose (LD_50_) was determined at the end of the second phase.

### Animals

The male Sprague-Dawley rats were procured from the Experimental Animal Unit of the Department of Animal production and Health of the Federal University of Technology, Akure, Ondo State, Nigeria; they were authenticated and used throughout the study. They were housed in plastic cages and maintained under standard natural photoperiodic condition of twelve hours of darkness and twelve hours of light (D:L; 12:12h dark/light cycle), at room temperature (25-32ºC) and humidity of 60-65%. The rats were fed with standard rat chow (Farm support Ltd, Akure, Ondo State) at a recommended dose of 100 g/kg, as per advised by the International Centre of Diarrheal Disease Research, Bangladesh (ICDDR, B) daily. Drinking water was supplied *ad libitum*. They were acclimatized for two weeks before commencement of the administration. The weights of the rats were documented at procurement, during the period of acclimatization, at commencement of administrations and once a week throughout the experiment period, using an electronic analytical and precision scale (CAMRY EK5055, Indian). All experimental procedures followed the recommendations provided in the "Guide for the Care and Use of Laboratory Animals", prepared by the National Academy of Sciences and Published by the National Institute of Health (NIH, 1985).

### Experiment Design and Animal grouping

Fourty healthy male adult (12-14 weeks old) Sprague-Dawley rats weighing 200-220g were used for this study. The rats were randomly divided into four groups (A, B, C and D) of ten (n=10) rats each. Group A, which served as control, were given distilled water 2ml/kg b.wt each, daily, for 28 days. Group B, C and D rats were administered 100, 300 and 500mg/kg b.wt, each, daily, respectively for 28 days. The extract was administered once daily for six days within a week throught oral gavage. After the last dosing all the rats were sacrificed by cervical dislocation.

### Animal sacrifice and sample collection

At the time of sacrifice the rats were first weighed and then sacrificed by cervical dislocation. The abdominal cavity was opened up throught a midline abdominal incision to expose the reproductive organs. The testes were excised and trimmed of all fat. Blood sample was collected throught cardiac puncture for hormonal assay. The hormone assay (testosterone, follicule stimulating hormone and lutenizing hormone) was carried out using the immunoassay method as discribed by Saalu *et al.* (2013) and Yakubu *et al*. (2008). The testes and epididymis from the rats were carefully dissected out and weight independently. The testes from each rat were exposed carefully and removed. They were trimmed free of the epididymides and adjoining tissue.

### Hematological analysis

The blood samples were collected through cardiac puncture into sample bottles tubes coated with ethylene diamine tetra-acetic acid (EDTA). The samples were immediately analyzed for hematological parameters using the automated Sysmex apparatus of the type 8999. The parameters analyzed included: Hemoglobin (Hb), Packed Cell Volume (PCV), Red Blood Cell Count (RBC), White Blood Cell Count (WBC).

### Semen Analysis

The rats were sacrificed by cervical dislocation. Orchiectomy was performed by open castration method. The testicle was exposed by incising the tunica vaginalis and the cauda epididymis was harvested. The cauda epididymis of rats in each of the experimental group were removed and minced thoroughly in a specimen bottle containing normal saline for a few minutes to allow the sperms to become motile and swim out from the cauda epididymis (Saalu *et al.,* 2008).

### Sperm count and motility studies

After 5 min incubation at 37ºC (with 5% CO_2_), the semen was then taken with a 1ml pipette, dropped on a clean slide, and covered with cover slips. The slides were examined under light microscopy for sperm motility (Saalu *et al.*, 2008) and with the aid of the improved Neubauer hemocytometer (Deep1/10mm LABART, Germany) counting chamber, as described by Pant & Srivastava (2003); the spermatozoa were counted under the light microscope. The counting was carried out in five thoma chambers.

### Sperm morphology

This was done as described by Saalu *et al.* (2013). The sperm morphology was evaluated with the aid of a light microscope at x400 magnification. The caudal sperm was taken from the original dilution for motility, and diluted 1:20 with 10% neutral buffered formalin (Sigma- Aldrich, Canada). In wet preparations using phase contrast optics, the spermatozoa were categorized. In this study a spermatozoon was considered morphologically abnormal if it had a rudimentary tail, round or detached head and was expressed as a percentage of morphologically normal sperm.

### Testicular histology preparation

The histology of the testes was done by modifying the method reported by Kayode *et al.* (2007). The organs were harvested and fixed in Bouin‘s fluid for 24h, after which it was transferred to 70% alcohol for dehydration. The tissues were washed through 90% and absolute alcohol and xylene for different durations before they were transferred into two changes of molten paraffin wax for 1 hour each in an oven at 65ºC for infiltration. They were subsequently embedded, and serial sections were cut using rotary microtome at 5 microns. The tissues were picked up with albumenized slides and left to dry on a hot plate for 2 min. The slides were dewaxed with xylene and washed with absolute alcohol (2 changes); 70% alcohol, 50% alcohol and then water for 5 min. The slides were then stained with Hematoxylin and Eosin. The slides were mounted in DPX. Photomicrographs were taken at an x100magnification.

### Data presentation and statistical analysis

The data was expressed as Mean±SEM. Statistical differences between the groups were evaluated by one way ANOVA, followed by Dunnets comparison test to compare between treated and control groups. Differences yielding *p*<0.05 were considered statistically significant. Statistical analyses of the data were performed using the GraphPad Prism 5 software.

## RESULTS

### Acute toxicity studies

During the experimental procedure, no deaths, no locomotor activity alteration, no piloerection or any other clinical signs of toxicity were observed in any of the groups in both phases, even at a dose of 5,000mg/kg. Therefore, LD_50_>5,000mg/Kg, indicating that the extract was safe and nontoxic.

### Phytochemical screening

The qualitative analysis of *Solanum nigrum* leaves shows the presence of flavonoids, tannins, nasunin, terpenoids, saponins, alkaloids, steroids, phytate, oxalate and proteins. There were also high contents of vitamins A, C, D, E, amino acid and folic acid. There was also folate and minerals such as Na, Ca, K, Mg, and P.

### Sperm count and sperm motility

In Table 1. The mean values of sperm count and sperm motility in the control group administered 2ml/kg normal saline orally per day was 53.1±3.0 and 56.8±2.8 respectively. The experimental group B that received 100 mg/kg b.wt of *Solanum nigrum* extract showed no significant increase in sperm count and sperm motility (*p*>0.05) when compared with the value of the control group. However, there was significant (*p*<0.05) increase in mean sperm count and motility for groups C and D, when compared to that of the control group.

**Table 1:** Effects of aqueous leave-extract of *Solanum nigrum* on the sperm profile of Adult Sprague-Dawley rats after 28 days of oral consumption.

Parameters	Groups			
	A (Control)	B (100 mg/kg)	C (300 mg/ kg)	D (500 mg/kg)
Sperm court (x 10^6^ m/L)	53.1±3.0	60.5±3.0	64.4±2.8*	71.7±3.7*
Sperm Motility	56.8±2.8	63.1±2.4	71.6±2.6*	78.9±3.7*
Progressivity	X_0_	X_0_	X_0_	X_0_
Normal Morphology (%)	75.9±3.2	79.7±3.2	87.2±3.7*	95.1±2.2*
Abnormal Morphology (%)	19.5±0.7	17.0±0.5*	10.5±1.2*	7.4±0.2*
Live /dead ratio livability (%)	71.8±2.4	79.5±2.3*	87.4±2.2*	95.3±2.1*

Values are expressed as Mean ± S.E.M n=10 in each group,

* represent significant dissimilarly from the control group at *p*<0.05.

One-Way ANOVA. X0: Rapid linear progressive motility

A: 2ml/kg b.wt of normal saline B: 100 mg/kg b.wt of *Solanum nigrum* extract. C: 300 mg/kg b.wt of *Solanum nigrum* extract. D: 500 mg/kg b.wt of *Solanum nigrum* extract

### Sperm progressiveness and sperm morphology

There was a significant (*p*<0.05) difference in sperm progressiveness across the group in a dose-dependent manner. The percentage number of normal sperm significantly increased across the groups, although there was a decrease in the percentage number of abnormal sperm across the groups (Table 1).

### Livability (Live /dead ratio)

There was significant increase in the percentage number of live/dead sperm across the group, when compared with content in the control group in a dose-dependent manner.

### Serum Testosterone Level, Follicle Stimulating Hormone and Luteinizing hormone

As shown in Table 2, the mean testosterone level of the Control group treated with 2 ml/kg normal saline was 1.67±0.10. The mean testosterone level of groups B, C and D (1.77±0.11, 1.87±0.12 and 1.97±0.13) showed no significant statistical difference when compared to the Control Group. There was a significant increase in the blood follicle stimulating hormone (FSH) mean value in Group B (0.18±0.01), when compared with the Control group. However, there was no significant increase in the FSH level of groups C and D. In addition, the luteinizing hormone (LH) mean value of group B treated with 100 mg/kg b.wt of *Solanum nigrum* extract was not significantly different when compared to the Control group. However, there was a significant increase in the mean LH levels of groups C and D (0.12±0.00 and 0.13±0.01), when compared with that of the Control group (0.09±0.01).

**Table 2:** Effect of aqueous leaves extract of Solanum nigrum onserumTestosterone, Follicule stimulating hormone and Leutenizing hormone of Adult Sprague- Dawley rats after 28days of oral consumption.

Parameters	Groups			
	A (2 ml/kg)control	B (100 mg/kg)	C (300 mg/ kg)	D (500 mg/kg)
Testosterone (ngm/L)	1.67±0.10	1.77±0.11	1.87±0.12	1.97±0.13
FSH (miu m/L)	0.16±0.01	0.18±0.01	0.20±0.01*	0.22±0.01*
LH (miu m/L)	0.09±0.01	0.10±0.00	0.12±0.00*	0.13±0.01*

Values are expressed as Mean ± S.E.M n=10 in each group,

* represent significant dissimilarly from the control group at P<0.05.

One-Way ANOVA. FSH: Follicle stimulating hormone, LH: Luteinizing hormone Miu: Milli international unit, ng: Nanogram. A: 2ml/kg b.wt of normal saline B: 100 mg/kg b.wt of *Solanum nigrum* extract. C: 300 mg/kg b.wt of *Solanum nigrum* extract D: 500 mg/kg b.wt of *Solanum nigrum* extract

### Hematological Parameters

Table 3, shows the results of *Solanum nigrum* aqueous leaf extract on Hb, PCV, RBC and WBC values in male Sprague-Dawley rats. The result showed that Hb count increased significantly (*p*<0.05) in groups B and D, but significantly (*p*<0.05) decreased in Group C. There was significant (*p*<0.05) increase in the PCV count mean values across the groups in a dose-dependent manner when compared with the Control group. In addition, the mean value of RBC and WBC showed a significant increase in all the treatment groups when compared with the Control group.

**Table 3:** Blood levels of some hematological indices in Sprague-Dawley rats following 28 days oral administration of Solanum nigrum.

Parameters	Groups			
	A (2 ml/kg)control	B (100 mg/kg)	C (300 mg/ kg)	D (500 mg/kg)
PCV (%)	70.64±0.80	72.55±0.60	80.49±1.41*	89.34±1.78*
Hb (g/dl)	66.52±1.02	74.68±1.54*	62.77±0.57**	71.65±1.37*
WBC Count (X10^6^ mL^-1^)	9.01±0.29	13.80±0.43*	18.28±0.45*	24.72±0.37*
RBC Count (10^6^/mm^3^)	10.57±0.25	11.41±0.17	14.62±0.27*	17.82±0.46*

Values are expressed as Mean ± S.E.M n=10 in each group

* Represent significant increased from the control group at *p*<0.05

** represent significant decreased from the control.

One-Way ANOVA. PCV: Packed cell volume Red Blood Cell Counts (RBC), White Blood Cell Counts (WBC), Hemoglobin Concentration (Hb),

A: 2ml/kg b.wt of normal saline B: 100 mg/kg b.wt of *Solanum nigrum* extract C: 300 mg/kg b.wt of *Solanum nigrum* extract. D: 500 mg/kg b.wt of *Solanum nigrum* extract

### Changes in body and organ weight

Table 4 depicts a significant (*p*<0.05) increase in the changes observed in the body, testis and epididymal weight of the animals across the group receiving the aqueous extract *Solanum nigrum* in a dose-dependent manner. However, increase in epididymal weight in the group administered with 100mg/kg of body weight was not significant when compared to the Control group.

**Table 4:** Effect of aqueous leaves extract of *Solanum nigrum* on body weight, Testis weight and Epididymis weight of Sprague-Dawley rats after 28days of oral consumption.

Parameters	Groups			
	A (2 ml/kg)control	B (100 mg/kg)	C (300 mg/ kg)	D (500 mg/kg)
Initial body weight (g)	212.4±2.51	213.2±1.82	208.1±1.39	213±1.82
Final body weight (g)	253.4±2.99	264.6±2.54*	276.9±2.25*	287.0±1.69*
Weight gain (g)	41.0±0.48	51.4±0.48	68.8±0.86	74.1±0.13
Testis weight (g)	1.74±0.03	1.80±0.01*	1.85±0.01*	1.92±0.01*
Epididymis weight (g)	0.40±0.01	0.41±0.01	0.43±0.00*	0.44±0.01*

Values are expressed as Mean ± S.E.M n=10 in each group

* represent significant dissimilarly from the control group at *p*<0.05

One-Way ANOVA.

A: 2ml/kg b.wt of normal saline B: 100 mg/kg b.wt of *Solanum nigrum* extract. C: 300 mg/kg b.wt of *Solanum nigrum* extract. D: 500 mg/kg b.wt of *Solanum nigrum* extract

### Testicular histology

**Figure 1:**
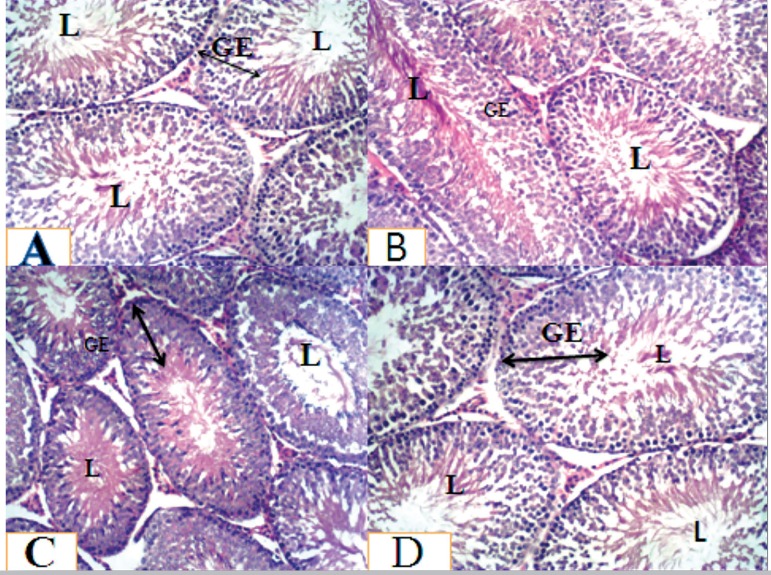
Histoarchitecture of the testes stained with H&E, X100. Group A, B, C and D rats showed normal cellular composition in their germinal epithelium (GE) with sperm cells in the lumen (L) and a normal interstitium. Also showed a normal spermatogenesis, better association and higher density of spermatogenic cells, complete maturation of germinal epithelium (GE) and lumen (L) contains full-mature spermatozoa.

The microphotograph of the testis of animals after 28 days of oral consumption of *Solanum nigrum* showed that groups A, B, C and D had a normal cellular composition in their germinal epithelium with sperm cells in the lumen and a normal interstitium. In addition, normal spermatogenesis, better association and higher density of spermatogenic cells, complete maturation of germinal epithelium and lumen contains full mature spermatozoa were evident in both the Control and Treated groups (Figure 1).

## DISCUSSION

The dual testicular function involves spermatogenesis and steroidogenesis. However, some conditions can interfere with spermatogenesis and reduce sperm quality and production. Several factors such as medication, chemotherapy, toxins, polluted air, lack of nutrients and vitamins can adversely affect spermatogenesis and sperm production (Mosher & Pratt, 1991). Normal spermatogenesis is set appropriately and the balance between cell proliferation and apoptosis is continuous (Allan *et al.,* 1992). In this present study, the oral administration of *Solanum nigrum* increased the spermatogenesis in Sprague-Dawley Rats with normal reproductive function. It can also be deduced that the high value of *Solanum nigrum* on spermatogenesis in our findings works via hypothalamus-pituitary-gonad axis. Therefore, the *Solanum nigrum* treatment can cause significant increase in serum FSH, LH and testosterone in infertile patients.

Testicular function is assessed, in part, by analyzing the spermatic indices including sperm count, motility, viability and morphology (Zinaman *et al*., 2000; Eliasson, 2003). Assessment of these parameters in the spermatozoa gives an indication of sperm quality and functionality. As normal sperm motility and count are vital for male fecundity (Zinaman *et al*., 2000), in our study we found that improvements in sperm count, sperm motility, percentage of normal morphology and percentage of the number of live sperm of the groups of animals administered with 100mg/kg, 300mg/kg and 500mg/kg of body weight after 28 days was due to oral consumption of aqueous crude extract of *Solanum nigrum*, when compared with the Control group. The result indicated that *Solanum* *nigrum* extract acts on the mitochondria in the body of the spermatozoon, where energy is being synthetized in the form of adenosine triphosphate, which increases sperm motility (Duke, 1997).

Oral consumption of *Solanum* *nigrum* could increase the glucose metabolism, leading to the production of pyruvate, which is known to be the preferred substrate, essential for the activity and survival of sperm cells (Egbunike *et al.,* 1986; Dua & Vaidya, 1996). In addition, the improved sperm parameters are also attributed to the amino acid content of *Solanum nigrum* (Fasuyi, 2006). Amino acids such as alanine, glycine, cystine and arginine, which are present in *Solanum* *nigrum* have been reported to preserve sperm cells and improve their motility (Bucak *et al.,* 2008). The findings from this study have shown that *Solanum nigrum* is rich in antioxidant constituents, such as flavonoids, saponins, vitamin E, vitamin C and vitamin A. Therefore, it is plausible to deduce that these rich antioxidant constituents of *Solanum nigrum* boosted the testicular non-enzymatic and enzymatic antioxidants to effectively scavenge free radicals, thus preventing lipid peroxidation. This finding is in concordance with the reports by Rodrigues *et al.,* 2005; Bansal & Bilaspuri, 2011. More so, vitamin E, a chain-breaking, non-enzymatic antioxidant also found in *Solanum nigrum* could inhibits lipid peroxidation in membranes by scavenging peroxyl (RO•) and alkoxyl (ROO•) radicals (Saleh & Agarwal, 2002). Furthermore, in our findings, phytochemical screening of *Solanum* nigrum revealed high values of vitamins A, C, D, E, amino acid and folic acid and also folate and minerals such as Na, Ca, K, Mg, P. Vitamin E supplementation has been found to increase fertilization rates, possibly by improving membrane integrity, reducing oxidative damage and lipid peroxidation potential (Geva *et al.,* 1996; Comhaire *et al.,* 2000). It was therefore deduced that vitamins A, C, D, E and flavonoids seen in *Solanum* nigrum promote spermatogenesis in Sprague-Dawley rats and is in accordance with reports by Aitken & Roman (2008).

The antioxidants in the aqueous extract of *Solanum* nigrum such as flavonoid and vitamins could enhance sperm production, sperm morphology, sperm survival and sperm function. Therefore, it supplied additional nutrients to the groups of rats that consumed *Solanum nigrum* extract over the Control group rats. In our findings, there were elevations in serum testosterone levels, follicle stimulating hormone and luteinizing hormone of the experimental groups treated with *Solanum nigrum, which* showed the positive effects of the *Solanum nigrum* extract in Sprague-Dawley rats. It has been reported that treatment with antioxidants enhances steroidogenesis by improving the primary effects of the leydig cells, endocrine function along with increased circulating testosterone secretions and stimulation of spermatogenesis (Saalu *et al.,* 2013; Prasad & Rajalakshmi, 1989). Hematological parameters showed that the hemoglobin (Hb) count increased significantly in the 100mg/kg and 500mg/kg body weight groups but there was significant decrease in Hb count of the group that received 300mg/kg per body weight. In addition, there was significant increase in the mean values of PCV count across the groups in a dose-dependent manner when compared with the Control group. *Solanum nigrum* is being used as a food supplement, condiment and beverages to cure or ameliorate several disease conditions in the general population. Moreover, our result showed increases in hemoglobin count (Hb) in male Sprague Dawley rats. The increase in Hb count may be an indication that the plant extract could boost blood production when consumed within certain limits. Similarly, our result also showed that PCV count significantly increased in the group treated with 300 mg/kg and 500mg/kg of body weight of *Solanum nigrum* extract, when compared with the Control group. This increase, however, may be a positive indicator in boosting blood parameters in anemic patients.

In addition, there was normal cellular composition in the germinal epithelium of the treated groups, with sperm cells in the lumen and a normal interstitium. No observable lesion in the testes histology in all the treated groups when compared with the control animals. This is in accordance with reports from Cody *et al.* (1986), Harborne & Williams (2000), that plants containing flavonoids are effective in preventing lesion, mainly due to their antioxidant properties. However, in all the treated groups, there was an observed increased in spermatogenic activity towards the lumen of the seminiferous tubule. This increased cellular activity happened from the basement membrane up to the lumen of the seminiferous tubules of the testes. The reduced number of primary spermatogonia cells evidenced this. This is an indication that they might have differentiated to the next level of spermatogenic cells. This was mainly due to the presence of potent antioxidants, like flavonoids, that scavenge free radicals and increase testosterone formation by the interstitial cells of Leydig (Saalu *et al*., 2007).

## CONCLUSION

In conclusion, the consumption of the aqueous crude extract of *Solanum nigrum* effectively improves testicular function, as evident in the histomorphology, steroidogenesis, spermatogenesis and hematological indices in rats. *Solanum nigrum* extract can, therefore, be used as a potential fertility herb in male disorders and as hematinic agents for the treatment of anemia.
